# Supplementary material for: Polymer Grafted Aluminum Nanoparticles for Percolative Composite Films with Enhanced Compatibility
Source: Polymers (Basel). 2019 Apr 8;11(4):638. doi: 10.3390/polym11040638 (PMC6523902; doi:10.3390/polym11040638)
Supplement: Supplementary file 1 [file polymers-11-00638-s001.pdf]

Supporting Information

Polymer Grafted Aluminum Nanoparticles for Percolative Composite Films with Enhanced Compatibility

Chenggong Yang[1], Chufarov Marian[2, 3], Jie Liu[1], Qi Di[1], Mingze Xu[4], Yunhe Zhang[1]*, Wei Han[2, 3]* and Kun Liu[1]*

[1]State Key Laboratory of Supramolecular Structure and Materials, Jilin University, Changchun, 130012, P. R. China.

[2]Jilin Supercapacitor Engineering Laboratory, College of Physics, Jilin University, Changchun, 130012, P. R. China.

[3]International Center of Future Science, Jilin University, Changchun, 130012, P. R. China.

[4]International Joint Research Center for Nanophotonics and Biophotonics, School of Science, Changchun University of Science and Technology, Changchun 130022, P. R. China.

TOC

Isolated Aluminum nanoparticles grafted with polystyrene can be doped into polystyre films with a much better compatibility compared to naked aggregated Al NPs, almost no voids are in the composite films, thus leading to higher dielectric constant and enhanced breakdown strength. This method can be widely used for fabricating dielectric composites with high energy density with low loss.

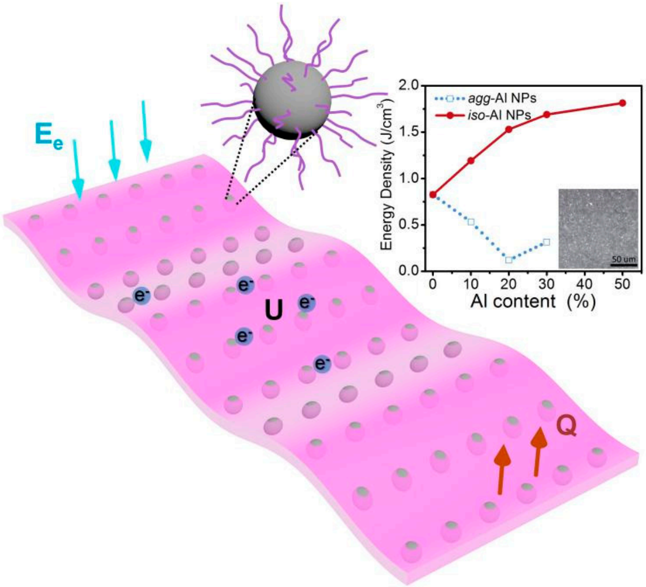

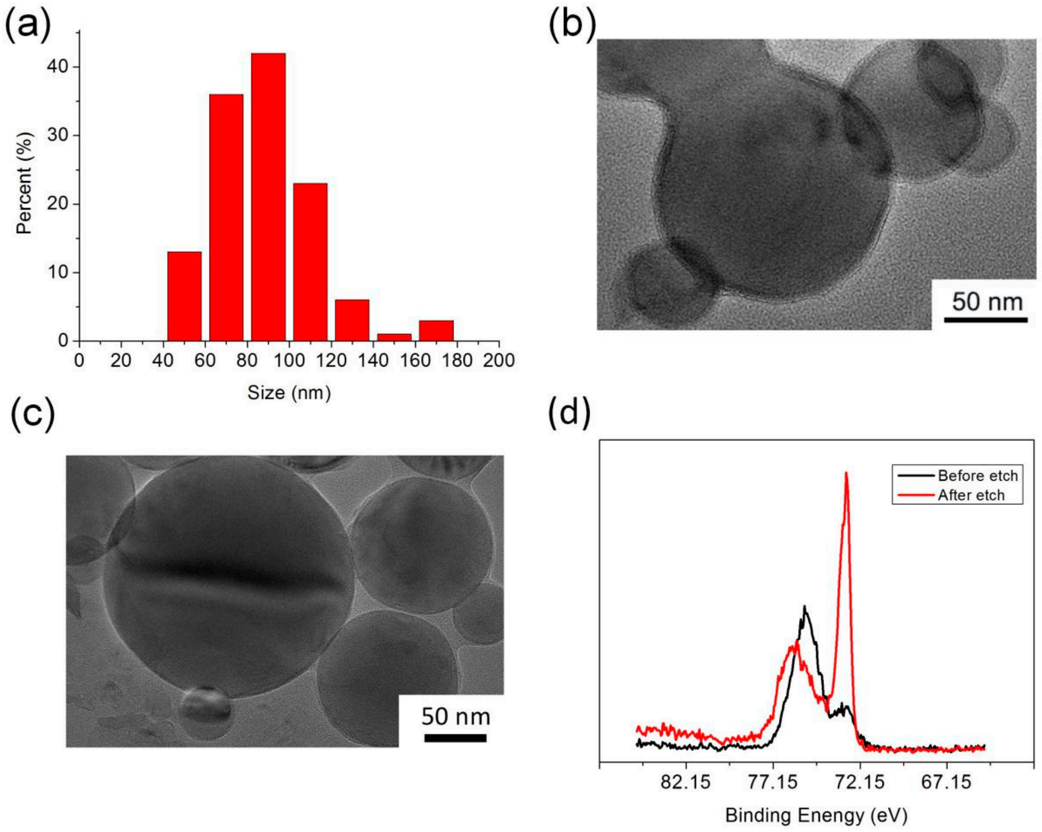

Figure S1. (a) Statistics histogram of the size of isolated Al NPs. High resolution TEM images of (b) aggregated Al NPs and (c) isolated Al NPs grafted with PS–COOH. (d) X-ray photoelectron spectroscopy spectra of Al 2*p* of aggregated Al NPs before and after etching with Ar plasma.

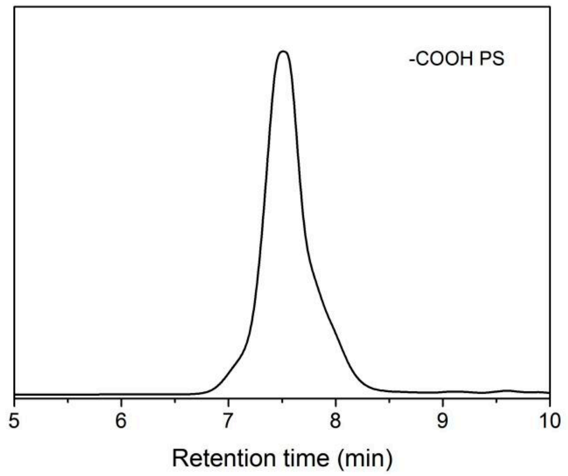

Figure S2. Gel permeation chromatography (GPC) elution curves of PS-COOH initiated by ACVA.

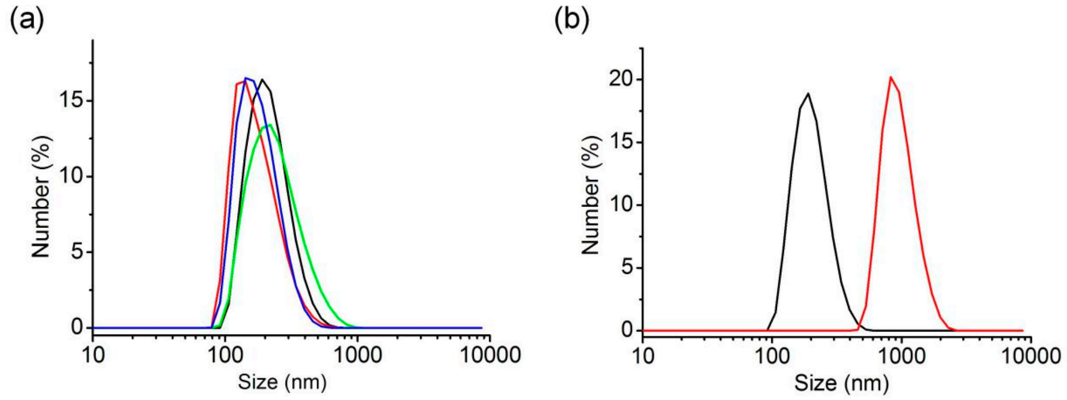

Figure S3. (a) DLS results of *iso*-Al NP@PS before (black line) and after purified by centrifugation for once (red line), twice (green line), and three times (blue line). (b) DLS results of *agg*-Al NPs (red line) and *iso*-Al NPs@PS (black line).

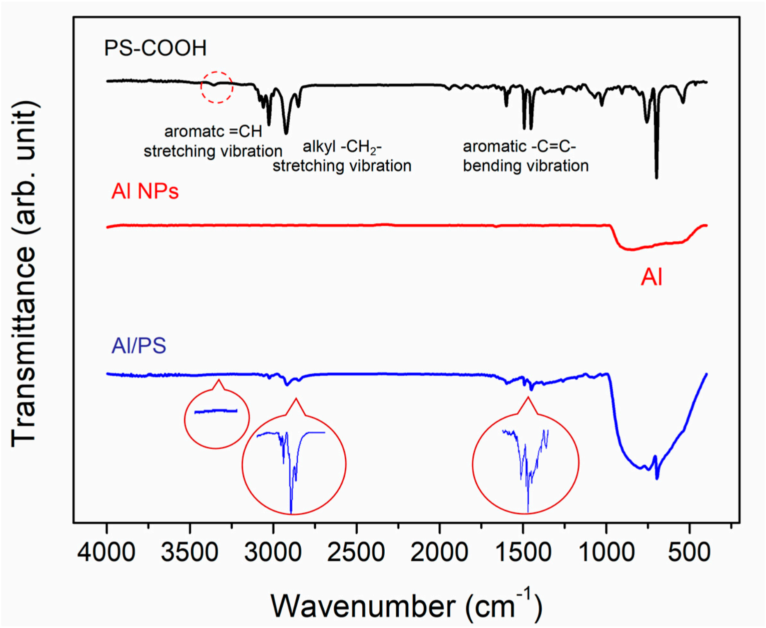

Figure S4. FTIR spectra of pure PS-COOH and Al NPs before and after grafting with PS-COOH.

In the FTIR spectra of PS-COOH, a relatively week peak at 3360 cm^{-1} corresponds to the -OH stretching vibration of the -COOH group of PS. After grafting on Al NPs, this peak at 3360 cm^{-1} disappeared, which indicate the -COOH group of PS was successfully reacted on the Al NPs surface.

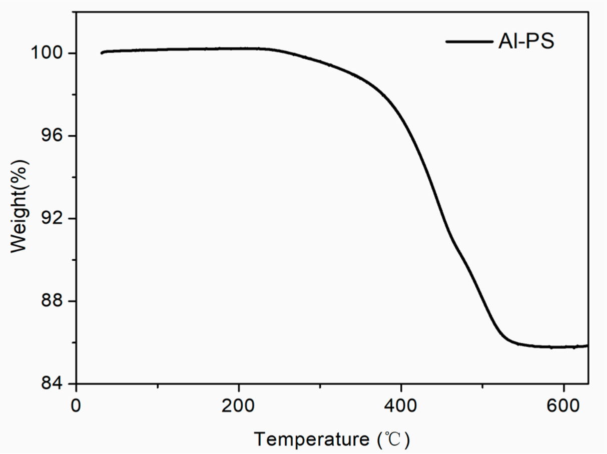

Figure S5. TGA curve of Al NPs grafted with PS-COOH.

Through the TGA result, we can calculate the grafting density of PS-COOH on Al NPS as following: the average radius (R_{Al}) of the Al NPs grafted with PS was mainly concentrated at 90 nm. The molecular weight of COOH-PS (MW_{PS}) was 21500 g/mol. The density of bulk Al (ρ_{core}) is 2.7 g/cm^3. N_A is the Avogadro number.

The weight fraction of PS (W_{PS}) and Al NP core (W_{Al}) were 14.2% and 85.8%, respectively, determined by the TGA measurement. The grafting density (σ) can be calculated with the following equation:

$$\sigma = \frac{\frac{4}{3}\pi {R_{Al}}^3 W_{PS}\rho_{core}N_A}{4\pi {R_{Al}}^2 W_{Al} MW_{PS}} = \frac{R_{Al}W_{PS}\rho_{core}N_A}{3\pi W_{Al} MW_{PS}}$$

$$= \frac{90\ \text{nm} \times 14.2\% \times 2.7\ \text{g/cm}^3 \times 6.02 \times \frac{10^{23} chains}{\text{mol}}}{3 \times 85.8\% \times 21500\ \text{g/mol}} = 0.12\ \text{chains/nm}^2$$

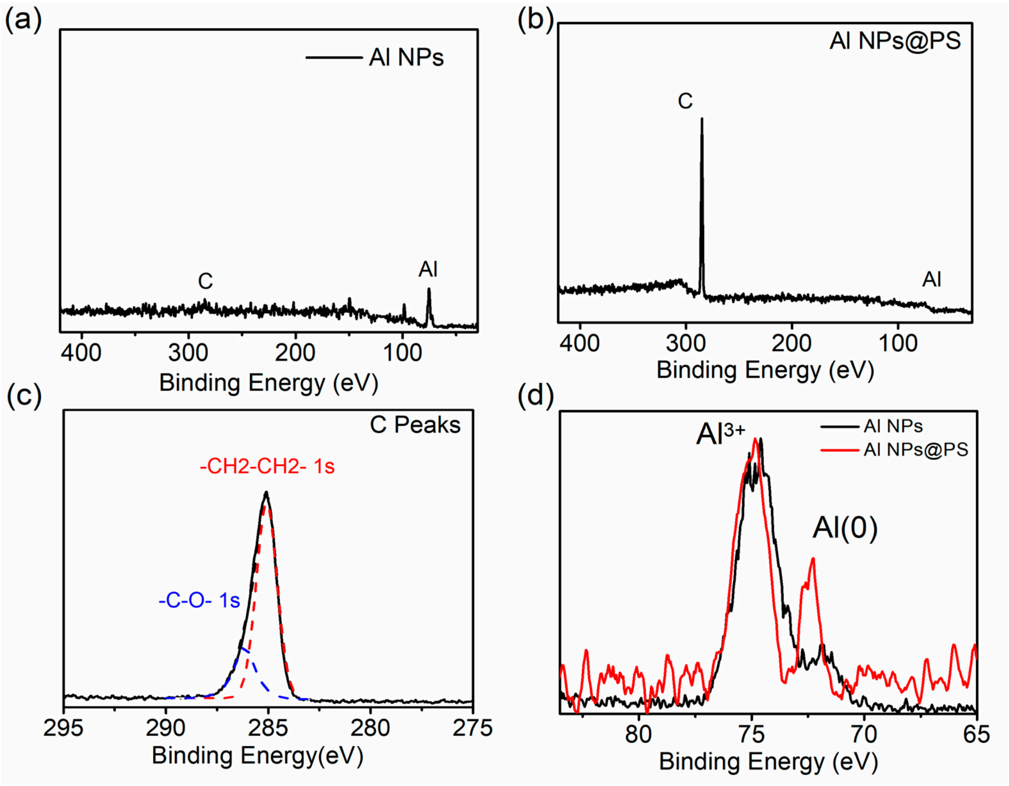

Figure S6. XPS spectra of Al NPs (a) before and (b) after grafting with PS. (c) C 1s orbital spectrum of Al NPs after grafting with PS, where the black solid line represents the original data of C 1s spectrum, the red and blue dash lines are for the fitting curves of -C-H2 and -C-O-, respectively. (d) Al 2p 3/2 orbital spectra of Al NPs before (black line) and after grafting with PS (red line).

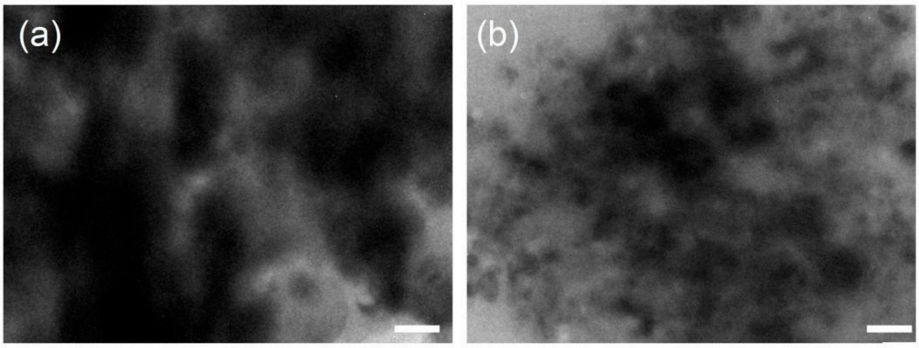

Figure S7. TEM images of (a) PS film doped with *agg*-Al NPs and (b) PS film doped with *iso*-NPs@PS. (scale bar is 200 nm)

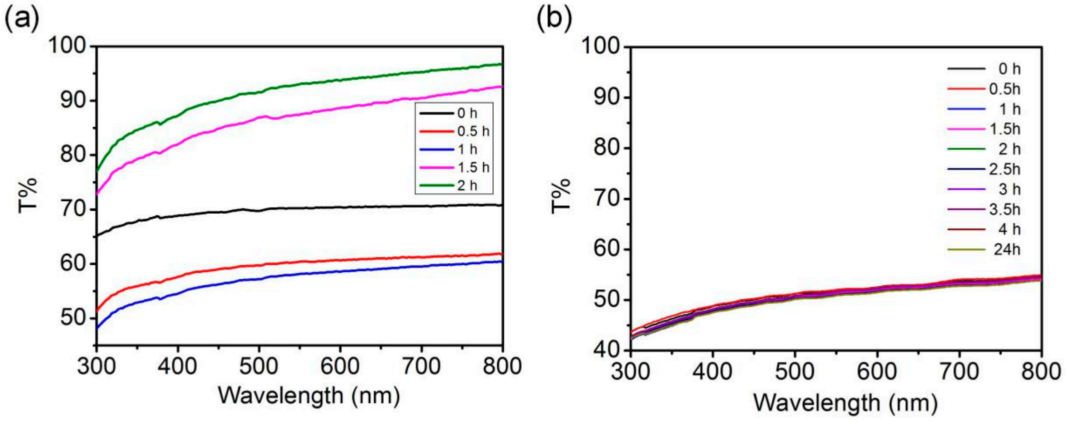

Figure S8. Moisture resistance of Al NP-PS composite films by testing the transmittance of the films. Transmittance spectra of (a) the PS film doped with *agg*-Al NPs and (b) the PS film doped with *iso*-Al NPs@PS.

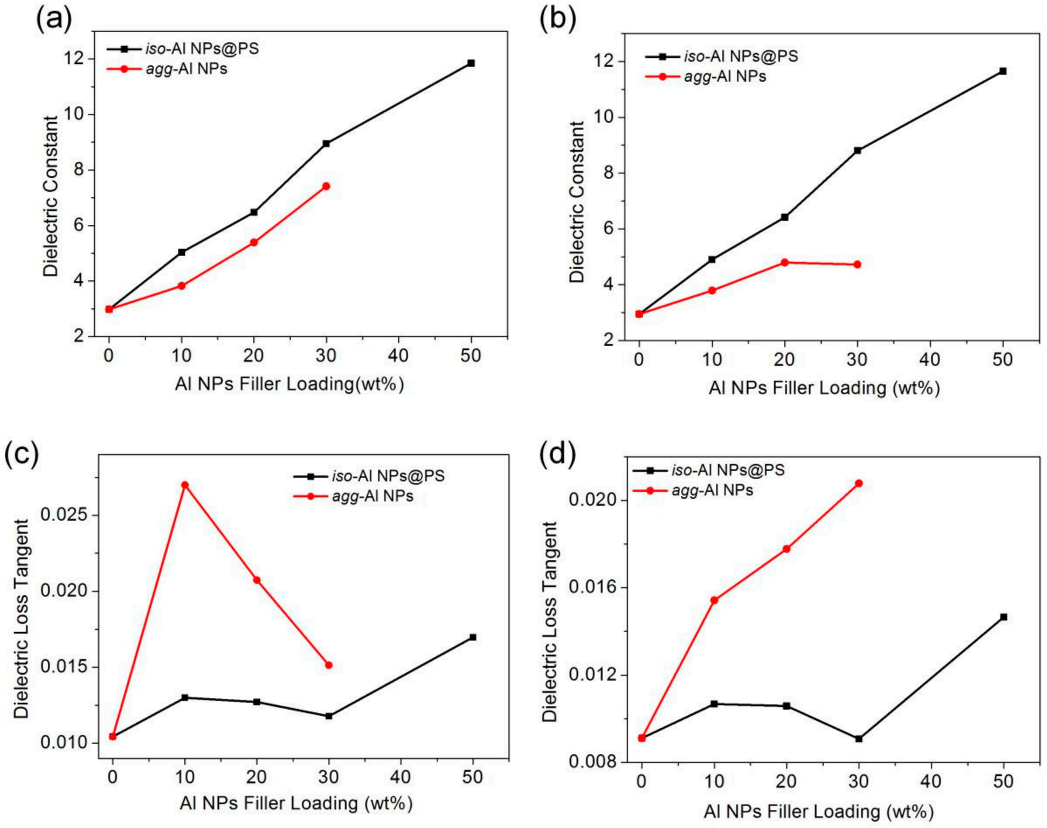

Figure S9. (a,b) Characteristic dielectric constant of PS films doped of *agg*-Al NPs and *iso*-Al NPs@PS at (a) 10^3 Hz and (b) 10^4 Hz with different filler loading. (c,d) The dissipation factor of PS films doped of *agg*-Al NPs and *iso*-Al NPs@PS at (c) 10^3 Hz and (d)10^4 Hz with different filler loading.

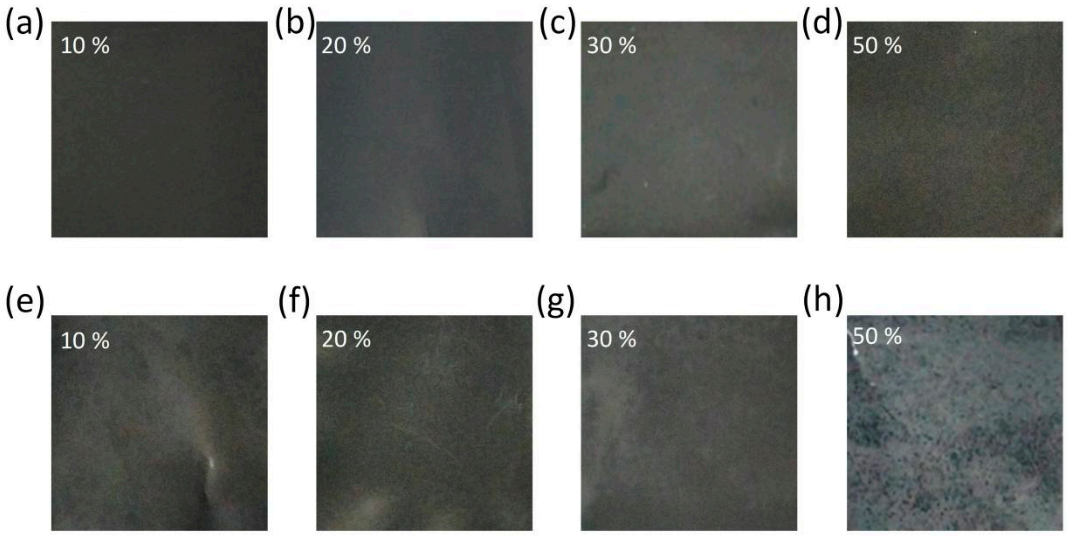

Figure S10. Optical photos of PS films doped with different filler loading of (a-d) *iso*–Al NPs@PS and (e–h) *agg*-Al NPs.

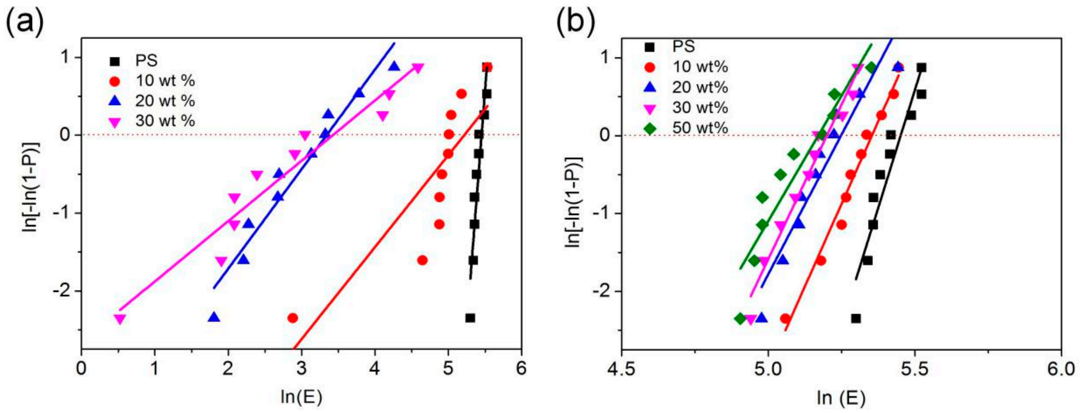

Figure S11. Two-parameter Weibull statistical distribution method for the characteristic breakdown strength values of (a) pure PS film and PS film doped with *agg*-Al NPs and (b) *iso*-Al NPs@PS with different Al NP filler loading as indicated in the legend.

Table S1. Characteristic Breakdown Strength (kV mm^{-1}) Calculated by Two-Parameter Weibull Statistical Distribution Method

Filler loading (wt %)/Samples	0 (pure PS)	10	20	30	50
Iso-Al NPs@PS	233.00	211.21	190.13	181.16	175.87
Agg-Al NPs	233.00	187.77	28.17	30.61	
